# Early vaccine effectiveness estimates against medically attended laboratory-confirmed influenza based on influenza surveillance, Beijing, China, 2024/25 season

**DOI:** 10.2807/1560-7917.ES.2025.30.7.2500084

**Published:** 2025-02-20

**Authors:** Ying Sun, Weixian Shi, Daitao Zhang, Chunna Ma, Zhaomin Feng, Jiaojiao Zhang, Dan Wu, Li Zhang, Jia Li, Wei Duan, Yingying Wang, Jiaxin Ma, Lu Zhang, Xiaodi Hu, Peng Yang, Quanyi Wang

**Affiliations:** 1 Beijing Key Laboratory of Surveillance, Early Warning and Pathogen Research on Emerging Infectious Diseases, Beijing Center for Disease Prevention and Control, Beijing, China; 2 Beijing Research Center for Respiratory Infectious Diseases, Beijing, China; 3 School of Public Health, Capital Medical University, Beijing, China

**Keywords:** influenza, vaccine effectiveness, early epidemic, A(H1N1)pdm09

## Abstract

We estimated early influenza vaccine effectiveness (VE) for the 2024/25 season in outpatients, in Beijing using a test-negative design. A(H1N1)pdm09 dominated (99.3%), all sequenced strains (n = 38) clustered in clade 6B.1A.5a.2a, and 37 of 38 antigenically similar to the vaccine strain. VE against any influenza virus infection was 48.5% (95% CI: 34.8–59.5) and 48.7% (95% CI: 35.1–59.7) against A(H1N1)pdm09. Vaccination in the current or previous season against any influenza showed a VE of 52.5% to 54.9%, compared to no vaccination in both seasons.

Timely early estimates of influenza vaccine effectiveness (VE) are crucial for formulating influenza prevention strategies and supporting World Health Organization (WHO) vaccine strain recommendations. The robust influenza surveillance system in Beijing, China, provides favourable conditions for estimating VE [1]. Typically, in Beijing, VE is estimated after the epidemic period, often after the month of April, limiting its timeliness for informing prevention strategies and WHO recommendations. Here, we used influenza surveillance data to deliver timely VE estimates using a test-negative design during the 2024/25 influenza epidemic's early phase.

## Data sources and study design

The Influenza Surveillance System in Beijing includes 40 sentinel hospitals and 19 network laboratories. Each week, oropharyngeal swab samples of at least 20 influenza-like illness (ILI) outpatients from each sentinel hospital are tested for influenza using real-time reverse transcription PCR (RT-PCR) assays. To ensure balanced ILI selection, approximately five patients are recruited daily per sentinel hospital, with sampling distributed throughout the day whenever possible. 

The ILI is defined as a measured or self-reported axillary temperature ≥ 38 °C with cough or sore throat, with symptom onset occurring within the past 10 days. Patients were classified as effectively vaccinated in current season if their vaccination date was at least 14 days before symptom onset. Those receiving at least one vaccine dose in the 2023/24 season were considered vaccinated for previous season. Vaccination status for both the current and previous influenza seasons was verified using the Immunization Planning Information Management System, which contains demographic and vaccination information, such as identification number, name, age, sex, types of influenza vaccines administered and immunisation date.

To estimate influenza VE, we employed a test-negative design. Cases were defined as ILI patients with PCR-confirmed influenza, while controls were ILI patients who tested negative. ILI patients were recruited between 28 October 2024 (week 44) and 12 January 2025 (week 2).

## Characteristics of the study population

From 28 October 2024 to 12 January 2025, a total of 8,775 ILI outpatients were recruited. Of these, 6,741 (76.8%) tested negative for influenza, while 2,034 (23.2%) tested positive. Among the positive cases, 2,020 (99.3%) were identified as A(H1N1)pdm09, 14 (0.7%) as A(H3N2) and 2 (0.1%) as B(Victoria) (Table 1).

**Table 1:** Descriptive characteristics of enrolled ILI patients, Beijing, China, week 44 2024 to week 2 2025 (n = 8,775)

Characteristics	A(H1N1)pdm09 n = 2,020^a^		A(H3N2) n = 14^a^		B(Victoria) n = 2		Influenza-positive cases n = 2,034		Test-negative controls n = 6,741		Total n = 8,775		p value^b^
	n	%	n	%	n	%	n	%	n	%	n	%	
Age in years, median (IQR)	37.0 (22.0–49.0)		32.0 (29.0–47.0)		27.0 (26.0–28.0)		37.0 (22.0–49.0)		26.0 (11.0–39.0)		29.0 (12.0–41.0)		< 0.001
**Sex**													
Male	987	48.9	6	42.9	1	50.0	992	48.8	3,098	46.0	4,090	46.6	0.026
Female	1,033	51.1	8	57.1	1	50.0	1,042	51.2	3,643	54.0	4,685	53.4	
**Resident area**													
Urban	914	45.2	5	35.7	0	0.0	919	45.2	2,911	43.2	3,830	43.7	0.11
Suburban	1,106	54.8	9	64.3	2	100.0	1,115	54.8	3,830	56.8	4,945	56.4	
**Comorbidities^c^**													
Yes	145	7.2	3	21.4	0	0.0	148	7.3	417	6.2	565	6.4	0.08
No	1,875	92.8	11	78.6	2	100.0	1,886	92.7	6,324	93.8	8,210	93.6	
**Pneumonia**													
Yes	68	3.4	1	7.1	0	0.0	68	3.3	501	7.4	569	6.5	< 0.001
No	1,952	96.6	13	92.9	2	100.0	1,966	96.7	6,240	92.6	8,206	93.5	
**Vaccination status in current season^d^**													
Vaccinated	123/1,984	6.2	1	7.1	0	0.0	124/1,998	6.2	1,000/6,444	15.5	1,124/8,442	13.3	< 0.001
Non-vaccinated	1,861/1,984	93.8	13	92.9	2	100.0	1,874/1,998	93.8	5,444/6,444	84.5	7,318/8,442	86.7	

ILI: influenza-like illness; IQR: interquartile range.

^a^ Includes two co-infections with A(H1N1)pdm09 and A(H3N2).

^b^ P values compare influenza-positive case and test-negative control participants. The p value for age is calculated using the Wilcoxon test, while p values for other factors are calculated using the Chi-square test.

^c^ Comorbidities are defined as the presence of at least one comorbidity, including chronic obstructive pulmonary disease, asthma, cardiovascular disease, diabetes, immunodeficiency or organ transplant, renal impairment, rheumatologic disease, neuromuscular disease, cirrhosis or liver disease, neoplasms, autoimmune diseases or haematological diseases.

^d^ Of the total, 8,442 participants (96.2%) have vaccination information recorded in the Beijing Immune Planning Information Management System, including 1,998 influenza-positive (98.2%) and 6,444 influenza-negative (95.6%) participants. We compared the case and control groups among individuals without vaccination records and found no statistically significant differences in age or sex.

Significant differences were observed between the two groups in terms of age, sex, comorbidity, pneumonia diagnosis and vaccination status (Table 1). The median age in the influenza-positive case group was higher (37.0 years) compared with the test-negative control group (26.0 years). In the case group, 48.8% were male, compared with 46.0% in the control group. Additionally, 7.3% of the case group had at least one comorbidity (vs 6.2% in the control group), while 3.3% of the case group had pneumonia, compared with 7.4% in the control group.

## Phylogenetic analysis

We randomly selected some samples of influenza strains during the 2024/25 season to assess their genetic clades and antigenic similarity with the vaccine strain. All sequenced influenza A(H1N1)pdm09 viruses (38/38) clustered within clade 6B.1A.5a.2a. Among them, three belonged to subclade 6B.1A.5a.2a.1, and 35 belonged to clade 6B.1A.5a.2a. All viruses were closely related to the reference vaccine strain, A/Victoria/4897/2022 (H1N1)pdm09-like virus (6B.1A.5a.2a.1) (Figure). Antigenicity analyses using hemagglutination inhibition (HI) assays confirmed that 37 of 38 viruses were antigenically similar to the egg-based vaccine strain.

**Figure:**
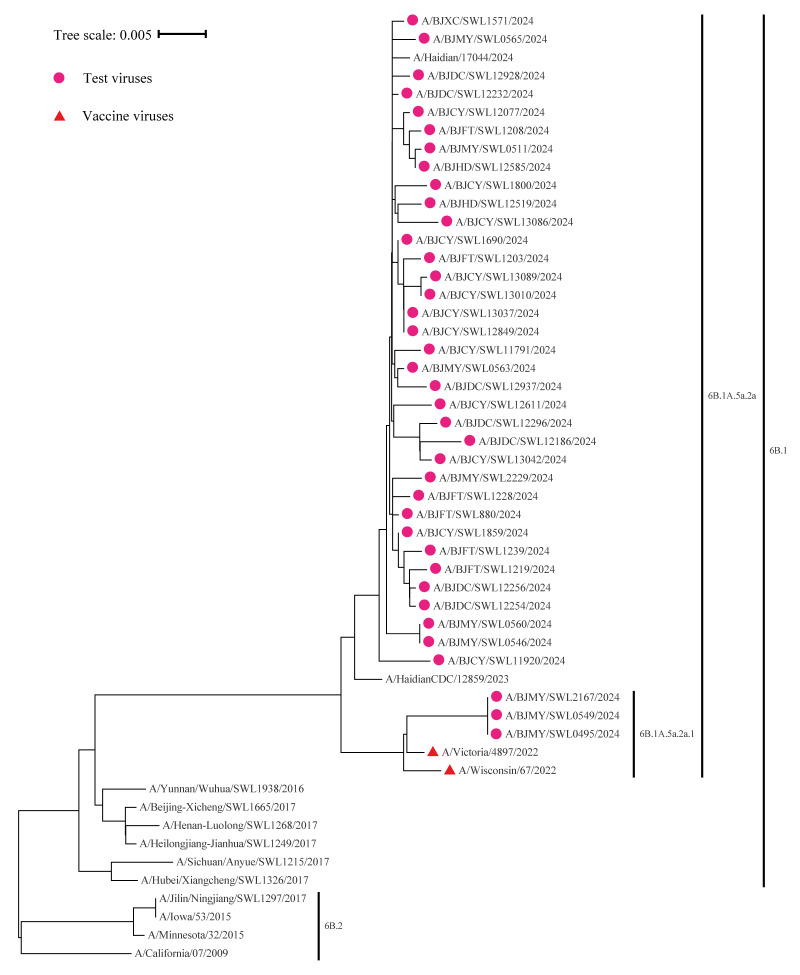
Phylogenetic analysis of the haemagglutinin (HA) gene of the influenza circulating A(H1N1)pdm09 virus strains (n = 38) compared to reference vaccine strains, Beijing, China, week 44 2024−week 2 2025

The PCR products were sequenced using the Illumina MiniSeq deep sequencing platform, and additional HA sequences (n = 14) were obtained from the Global Initiative for Sharing All Influenza Data (GISAID) [21]. Nucleotide sequences were assembled and aligned using MEGA software (version 5.0).

## Vaccine effectiveness estimates

During the early 2024/25 season up to week 2 2025, the vaccination coverage was significantly lower in the case group (124/1,998; 6.2%) compared with the controls (1,000/6,444; 15.5%), regardless of comorbidity or pneumonia status (Table 2). This trend was also consistent across all age groups. Odds ratios (ORs) for vaccination status were estimated using logistic regression. If the sample size within a stratum was too small, penalised logistic regression was applied to assess potential small-sample bias compared with the original ORs. The VE was calculated as (1−OR) x 100%. The adjusted overall VE against influenza was estimated at 48.5% (95% CI: 34.8–59.5) (Table 2).

**Table 2:** Influenza vaccine effectiveness estimated using the test-negative design, Beijing, China, week 44 2024−week 2 2025 (n = 8,442)

Characteristics	Influenza-positive cases		Test-negative controls		Vaccine effectiveness			
	n/total	%	n/total	%	Crude VE estimates (%)	95% CI	VE estimates from adjusted OR^a^ (%)	95% CI
**Overall**	124/1,998	6.2	1,000/6,444	15.5	64.0	56.4 to 70.5	48.5	34.8 to 59.5
**Age in years**								
0–5	11/150	7.3	63/566	11.1	36.8	−18.5 to 69.2	57.9	15.2 to 80.6
6–17	91/264	34.5	795/1,844	43.1	30.6	9.3 to 47.2	34.9	11.9 to 52.1
18–59	6/1,319	0.5	77/3,432	2.2	80.1	57.9 to 92.3	83.9	64.1 to 94.0
≥ 60	16/265	6.0	65/602	10.8	46.9	8.7 to 70.8	52.9	7.6 to 76.8
**Comorbidities^b^**								
Yes^c^	3/147	2.0	29/412	7.04	72.5	21.0 to 93.5	77.4	24.8 to 95.1
No	121/1,851	6.5	971/6,032	16.1	63.5	55.8 to 70.2	47.2	32.9 to 58.7
**Pneumonia**								
Yes^d^	2/68	2.9	58/495	11.7	77.2	24.4 to 96.3	65.3	−41.3 to 94.9
No	122/1,930	6.3	942/5,949	15.8	64.1	56.5 to 70.6	47.8	33.7 to 59.1
**Subtype**								
A(H1N1)pdm09	123/1,984	6.2	1,000/6,444	15.5	64.0	56.5 to 70.5	48.7	35.1 to 59.7

CI: confidence interval; OR: odds ratio; VE: vaccine effectiveness. Most (99.8%) influenza vaccinations in Beijing during the 2024/25 season are inactivated influenza vaccines, with 87.1% being trivalent and 12.9% quadrivalent.

^a^ The overall VE against influenza is obtained by adjusting for age group (0–5, 6–17, 18–59, ≥ 60 years), sex, resident area, calendar week (week 44 in 2024 to week 2 in 2025), comorbidities, and pneumonia diagnosis. VE by age group is adjusted for sex, resident area, calendar week, comorbidities and pneumonia diagnosis. VE by comorbidity status is adjusted for age group, sex, resident area, calendar week and pneumonia diagnosis. VE by pneumonia status is adjusted for age group, sex, resident area, calendar week and comorbidities. VE against influenza subtype A(H1N1)pdm09 is adjusted for age group, sex, resident area, calendar week, comorbidities and pneumonia diagnosis.

^b^ Comorbidities are defined as the presence of at least one comorbidity, including chronic obstructive pulmonary disease, asthma, cardiovascular disease, diabetes, immunodeficiency or organ transplant, renal impairment, rheumatologic disease, neuromuscular disease, cirrhosis or liver disease, neoplasms, autoimmune diseases or haematological diseases.

^c^ The adjusted VE using penalised logistic regression (Elastic Net penalty with LASSO and Ridge each accounting for 50%) for participants with comorbidities was 73.7%.

^d^ The adjusted VE using penalised logistic regression for participants with pneumonia was 60.7%.

The adjusted VE against A(H1N1)pdm09 was 48.7% (95% CI: 35.1 to 59.7). Among age groups, adjusted VE was highest in the 18–59-year age group, with estimates of 83.9% (95% CI: 64.1–94.0), and adjusted VE in the 0–5, 6–17, and ≥ 60-year age groups were 57.9% (95% CI: 15.2–80.6), 34.9% (95% CI: 11.9 to 52.1), 52.9% (95% CI: 7.6 to 76.8), respectively. In individuals with comorbidities, the adjusted VE was 77.4% (95% CI: 24.8 to 95.1). Vaccination in either the current or previous season provided protection. The adjusted VE was 54.9% (95% CI: 40.1 to 66.3) for those vaccinated in both seasons, 52.5% (95% CI: 32.2 to 67.3) for those vaccinated only in the current season, and 53.3% (95% CI: 36.6 to 66.0) for those vaccinated only in the previous season (Table 3).

**Table 3:** Influenza vaccine effectiveness based on vaccination status over two consecutive seasons, Beijing, China, 2023/24 and 2024/25 (n = 8,442)

Vaccination status	Influenza-positive cases n = 1,998		Test-negative controls n = 6,444		Vaccine effectiveness for all influenza			
	n	%	n	%	Crude VE		VE estimates from adjusted OR^a^	
					VE	95% CI	VE	95% CI
Unvaccinated	1,813	90.7	4,916	76.3	Reference		Reference	
Vaccinated (2023/24 only)	61	3.1	528	8.2	68.7	59.3 to 76.3	53.3	36.6 to 66.0
Vaccinated (2024/25 only)	45	2.2	311	4.8	60.8	46.7 to 71.8	52.5	32.2 to 67.3
Vaccinated (both seasons)	79	4.0	689	10.7	68.9	60.8 to 75.7	54.9	40.1 to 66.3

CI: confidence interval; OR: odds ratio; VE: vaccine effectiveness.

^a^ VE against influenza is adjusted for age group, sex, resident area, calendar week, comorbidities, and pneumonia diagnosis.

Most (99.8%) influenza vaccinations in Beijing during the 2023/24 and 2024/25 season are inactivated influenza vaccines. In the 2024/25 season, 87.1% were trivalent, and 12.9% were quadrivalent, while in the 2023/24 season, 57.6% were trivalent, and 42.4% were quadrivalent.

## Discussion

In Beijing, the free influenza vaccination policy implemented since 2007 for older residents (≥ 60 years) and school students (6–17 years) has led to coverage of around 15% and 50%, respectively [1]. Although the overall rate (around 10%) is lower than in European countries (around 20% in 2021) and the United States (42% in 2021) [2-5], it is notably higher than China's overall coverage (1.9–2.5%) [6,7]. Our study found a moderate VE (48.5%) against medically attended laboratory-confirmed influenza, similar to the interim finding of Canada (53%) [8]. In comparison, VE in Beijing was below 45% in the previous three seasons, while in Europe, it ranged from 30% to 53% [9-11], and in the United States, it varied between 19% and 48% over the past 10 seasons [12-14]. The VE was consistent across various subgroups, including for A(H1N1)pdm09, all age groups (except 6–17) and individuals with different comorbidities.

Several factors likely contributed to observed VE. The dominant circulation of A(H1N1)pdm09 and its close match with the vaccine strain were primary reasons. Over 95% of circulating influenza viruses were A(H1N1)pdm09, and genetic sequencing analysis suggests that most circulating A(H1N1)pdm09 viruses are closely related to A/Victoria/4897/2022 (H1N1)pdm09-like virus, though the sample size was limited. Antigenicity analyses showed most A(H1N1)pdm09 viruses were similar to the 2024/25 vaccine strain, consistent with the report by the China CDC this season [15]. Similarly, during Australia's 2024 season, 46% of circulating influenza viruses were A(H1N1)pdm09, of which 98.7% matched the vaccine strain, and were identical to the northern hemisphere vaccine strain, resulting in a reported VE of 60% [16]. A(H1N1)pdm09, the dominant strain in China in the 2024/25 season, is also circulating in Europe (in week 1 2025) and North America (in week 52 2024), with most viruses in clade 6B.1A.5a.2a or sub-clades [15,17,18]. Early VE estimates from Beijing can provide valuable references for these regions.

Several points warrant attention. Firstly, the predominant circulating subtype in Beijing during the 2024/25 season was influenza A(H1N1)pdm09; this is the first time in the last decade that this influenza subtype has been predominant. If the circulating subtypes shifts to A(H3N2) in subsequent seasons, with only 56% antigenically similar to the A/Thailand/8/2022 (H3N2)-like vaccine strain, a lower future VE may be expected [15]. Secondly, the VE in the 6–17-year age group was relatively lower compared with other age groups, a pattern that was also observed in the previous season [14]. One possible explanation for this phenomenon is repeated vaccination [19,20]. Over the past three influenza seasons, influenza vaccination coverage among school-aged children in Beijing exceeded 40%, the highest among all age groups. As a result, many children received repeated vaccinations during this period. Given that the A(H1N1)pdm09 vaccine strain in the 2022/23 season was A/Victoria/2570/2019, prior vaccination and infection may have led to negative interference with responses against the circulating subtype. Thirdly, compared to no vaccination in both seasons, vaccination in the previous season only showed moderate VE, suggesting the similarity between the vaccine composition in the previous season (A/Victoria/4897/2022 (H1N1)pdm09-like virus) and the circulating strains in current season potentially contributing to year-long protection.

A higher VE was observed among working-age adults in Beijing, providing direct protection against influenza that benefits both their health and the economy. Because this age group may be more socially active, their vaccination could contribute to indirect protection for their families, colleagues, and service recipients. To enhance this impact, we have included medical workers, teachers and long-term care facility workers in the free influenza vaccination policy in Beijing since September 2024 and encourage more adults to get vaccinated before the influenza season.

This study has some limitations. Participants were not randomly selected. To minimise bias, ILI patients among outpatients were selected with even distribution by day. The comorbidity status was self-reported, potentially introducing information bias. In addition, sequencing and antigenic analysis of only 38 of 1,783 A(H1N1)pdm09 strains may limit representativeness. The circulating strains could still change during the season, potentially influencing VE estimates.

## Conclusion

Our study showed moderate VE against medically attended laboratory-confirmed influenza, especially A(H1N1)pdm09, during the early 2024/25 influenza epidemic up to week 2 of 2025 in Beijing, China. These estimates provide timely insights for public communication and evidence for the World Health Organization's vaccine strain recommendations.
